# Audiological outcomes after revision stapes surgeries: a systematic review

**DOI:** 10.1007/s00405-024-08741-7

**Published:** 2024-06-05

**Authors:** László Székely, Imre Uri, Ágnes Luka, Anita Gáborján, László Tamás, Gábor Polony

**Affiliations:** 1 Department of Oto-Rhino-Laryngology, Head and Neck Surgery, Semmelweis Egyetem (Semmelweis University), Szigony Utca 36, Budapest, 1083 Hungary; 2 Department of Voice, Speech and Swallowing Therapy, Faculty of Health Sciences, Semmelweis University, Budapest, Hungary

**Keywords:** Otosclerosis, Stapes, Stapedotomy, Otologic surgical techniques, Systematic review

## Abstract

**Purpose:**

Revision stapes surgery is a challenging procedure performed in relatively small numbers compared to other middle ear procedures. Despite numerous data on hearing results of different middle ear surgeries, the audiological standards for successful outcome of this procedure are still not clarified. On the basis of well-documented data, we wanted to determine what the expected audiological results and complications are after revision stapes surgery in order to set a realistic threshold for surgical success.

**Methods:**

After the protocol registration in the PROSPERO database, a systematic review was performed in multiple databases (PubMed, Cochrane, Web of Science, Scopus, ScienceOpen, ClinicalTrials.gov, Google Scholar) according to PRISMA guidelines. Twelve articles were reviewed according to the inclusion criteria. A total of 1032 cases were obtained for evaluation. A modified version of Newcastle–Ottawa Scale (NOS) was used to assess publication quality.

**Results:**

Average air–bone gap (ABG) gain was 17.3 dB, average air conduction (AC) gain was 17.5 dB. The average postoperative air–bone gap was 11.1 dB. The postoperative ABG distribution was the following 0–10 dB: 53.3%, > 10–20 dB: 28.2%, > 20 dB: 18.5%. SNHL as a surgical complication was described in a total of 17 cases (1.6%), no equilibrium disorder was reported.

**Conclusion:**

The pooled data suggest that revision stapes surgery is an effective solution after failure of previous stapes surgery. However, the results are clearly inferior to those of primary stapedotomies. Hence, we need to apply different expectations and use different standards in the indication and evaluation of this type of surgery.

**Supplementary Information:**

The online version contains supplementary material available at 10.1007/s00405-024-08741-7.

## Introduction

The history of modern stapes surgery began in 1956 when Shea [1] performed the first stapedectomy with proper reconstruction of the ossicular chain. Since then, stapes surgery has made great progress and by the beginning of the twenty-first century, in Shea’s words, it “became so successful, the problem has now largely disappeared” [2]. Today, it is truly a flawless procedure at first glance, with high success rates, but in 3–13% of the cases [3], the results after primary surgery are not sufficient and these are the cases where problems are “reappearing”. The results of revision surgery appear to be inferior to primary surgery [4], for several reasons, some of them may be due to previous surgery. The impact of the previous procedure on the success of the revision varies. The most common and hardly reversible problem appears to be the formation of intratympanic adhesion [5, 6], which makes surgery challenging, while other issues such as prosthesis dislocations could be resolved with better results. The diversity of these problems leads to different outcomes. While expectations for primary stapes surgery are quite clear and the standards for success are well established, these standards are lacking in revision cases. According to the literature, a postoperative ABG of 10 dB or less is considered as success in stapes surgery [7–9]. In revision cases, the situations the surgeons have to deal with are different, and expectations before surgery are not as straightforward as in primary stapedotomy. The question is what level of hearing outcomes can be expected after revision stapes surgeries. The results of revision stapes surgery have been the subject of several publications, but individually the number of cases and the quality of data are limited. Our goal was to collect well-documented data from these previous publications to provide information to better understand the outcomes of revision stapedotomy. This information will lead to more realistic expectations, which is necessary for making appropriate counselling and indications, and it is useful in defining the standards of surgical success.

## Materials and methods

The Preferred Reporting Items for Systematic Reviews and Meta-analysis.

(PRISMA) guidelines were used to report the results.

*Eligibility criteria*: The PICO search strategy protocol was used, and the protocol for this study was registered at PROSPERO (ID: CRD42022242662). Patients (P) aged at least 16 years, with a diagnosis of stapes fixation (clinically, or surgically diagnosed), and who had undergone previous stapes surgery without the presence of osteogenesis imperfecta, congenital middle ear or inner ear deformity were involved. Only revision stapes surgeries (I) were involved in the investigation. Publications with inadequate surgical methodology were excluded. Due to the original question (C), the comparison was not applicable. The examined outcome (O) was postoperative average audiometry results measured at least 12 months after surgical intervention and postoperative morbidity. Only measurements according to the AAO HNS reporting guidelines with a follow-up of at least 12 months were included. Meta-analyses, systematic reviews, randomized controlled Trials, research studies or articles, case reports or series were all included in the search. Research reports or other grey literature and clinical practice guidelines were excluded.

*Search and selection*: A systematic literature search was performed in multiple databases: PubMed, Cochrane, Web of Science, Scopus, ScienceOpen, ClinicalTrials.gov, Google Scholar dating from January 1st 1991 to January 1st 2021, using the following search strategy: ("revision") AND ("stapes" OR "stapedotomy" OR "stapedectomy" OR "otosclerosis") Only English literature was included. The result studies were imported to EndNote X9.1 citation manager software (Thomson Reuters). Study selection was performed by two authors L.Á. and SZ.L. under the supervision of the senior author P.G..

*Data extraction and outcome measurement*: Data extraction was performed by two independent reviewers under the supervision of the senior author. Data were transferred to an Excel sheet, author, year of publication, study design, demographics, number of interventions, follow-up time, and specific outcome: pre- and postoperative PTA levels, complications, type of prosthesis, and surgical approach. Audiological results such as AC, ABG, BC Hearing Gain, ABG Gain, and averages were expressed in dBs. The mean of the thresholds at frequencies 0.5, 1, 2, and 3 or 4 kHz was used to form a four-tone pure-tone average. The pooled data were narratively reviewed and compared to former literature data on stapes surgery outcomes.

*Risk of bias (ROB) assessment*: Assessment was performed by the three authors in the same setting. The Modified Newcastle‐Ottawa Scale (NOS) was used in all cases, as all the involved manuscripts were nRCTs.

## Results

**Fig. 1:**
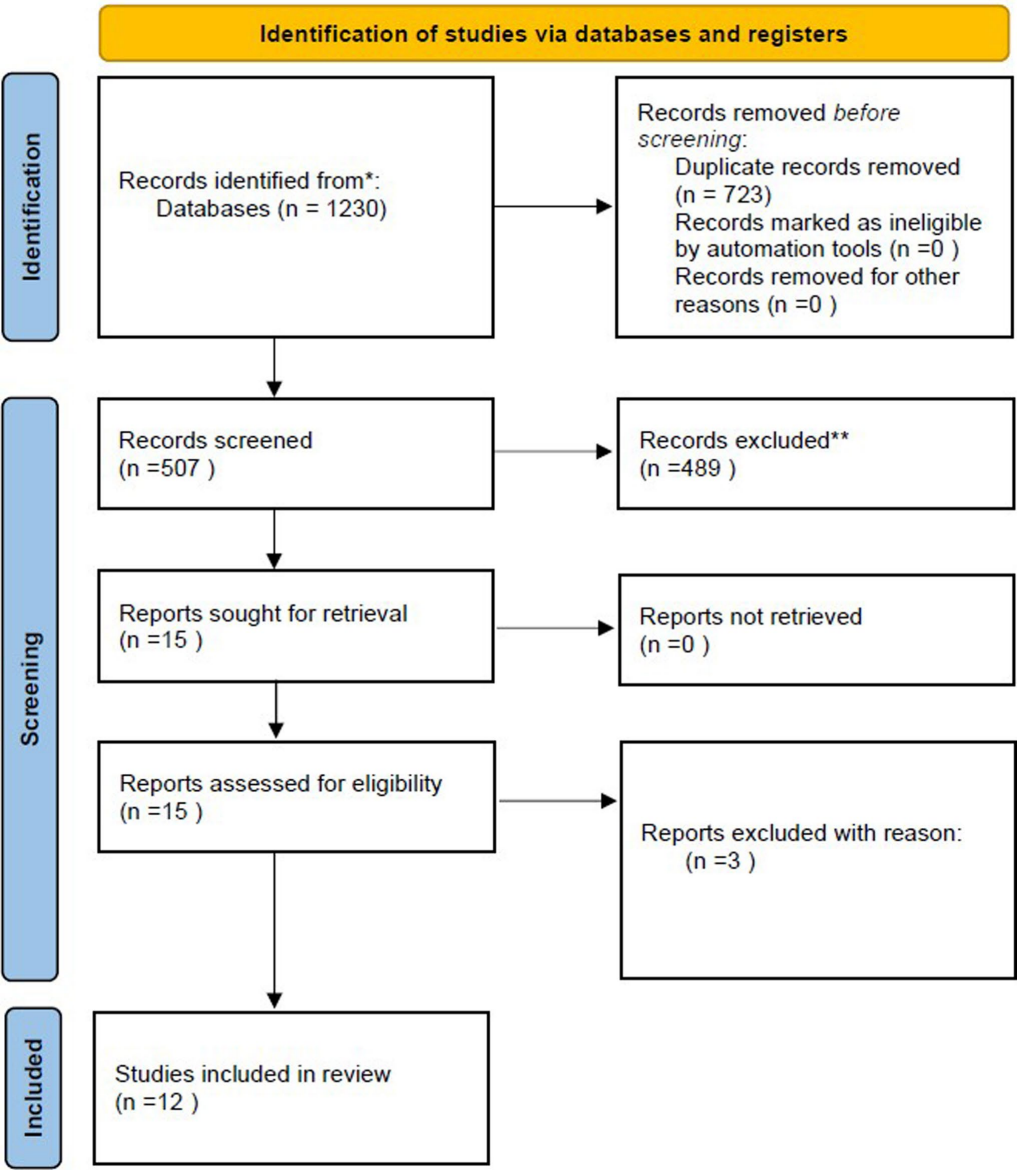
PRISMA flow diagram

*Study selection*: The search and selection process is shown in the PRISMA flow diagram (Fig. 1). After the first search, 1230 papers were found. After duplications were removed, 507 manuscripts were screened by title, abstract and full text. Fifteen publications met the eligibility criteria, of which three [10–12] were removed due to insufficient data after full-text screening. Twelve studies were included in the final review process[13–24], which represents 1032 cases. Article characteristics are shown in Table 1. Risk of bias assessment according to the modified version of NOS can be found in Supplement 1.

**Table 1:** Article characteristics

Author	Year	Study desing	Country/Number of centers	Number of cases	Mean follow-up time
E. Vartiainen	1992	Retrospective clinical study	Finland/1	45	7.6 years
W. Han	1997	Retrospective clinical study	USA/1	74	18.2 months
L. Krieger	1998	Retrospective clinical study	USA/2	83	1–20 years
W. Lippy	2002	Retrospective clinical study	USA/1	240	6.7 years
R. Puxeddu	2005	Retrospective clinical study	Italy/1	44	35 months
G. Babighian	2009	Retrospective clinical study	Italy/2	78	28 months
P. Schmid	2009	Retrospective clinical study	Switzerland/1	172	1 year
M. Özüer	2012	Retrospective clinical study	Turkey/1	84	19 months
J. Skrivan	2014	Retrospective clinical study	Czech Republic/1	43	1 year
M. Ghonim	2017	Retrospective clinical study	Egypt/1	36	23 months
D. Bernardeschi	2018	Retrospective clinical study	France/1	102	15 months
U. Fisch	2001	Retrospective clinical study	Switzerland/1	71	1 year

*Patients and procedure*: According to the pooled data, the average age was 47.8 years, ranging from 16 to 88 years. The female to male ratio was 61–39%.

**Fig. 2:**
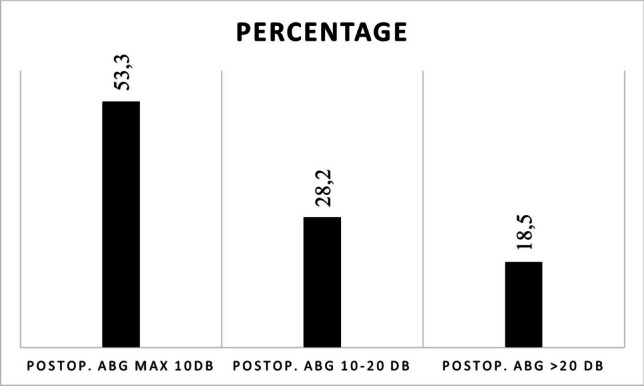
Postoperative ABG closure distribution in 10 dB bins

*Hearing results*: Pre- and postoperative AC levels were available in 484 cases. Mean preoperative AC level was 61.1 dB, mean postoperative AC was 43.6 dB, which means an average of 17.5 dB hearing gain after the procedure. Pre- and postoperative ABG levels were available in 571 and 712 cases, respectively. The average preoperative ABG was 28.4 dB, postoperative ABG was 11.1 dB, this represents an average ABG gain of 17.3 dB (Table 2). Postoperative ABG distribution in 10 dB bins was available in 875 cases. ABG closure of 10 dB or less was achieved in 53.3% of the cases, whereas in 28.2%, ABG was between 10 and 20 dB, and in 18.5% of the cases, postoperative ABG remained 20 dB or higher (Fig. 2).

**Table 2:** Pre- and postoperative hearing results

	Mean threshold (dB)	Mean gain (dB)	No. of cases
Preop. AC	61.1	17.5	484
Postop. AC	43.6		484
Preop. ABG	28.4	17.3	571
Postop. ABG	11.1		712

### Surgical technique and findings

Numerical data about stapes prosthetics was available in 10 publications. The type of the revised prosthesis was available in 278 cases. Retrieved prosthesis showed a wide range of variation with a relatively high number of wire type prosthetics, and autologous reconstructions (Table 3). Data about newly inserted prosthetics was available in 678 cases. In the majority of the cases (59%) classic piston type prosthetics was used, autologous and wire type solutions were used in minimal numbers. Malleovestibulopexy was performed in 23% of the cases and in 4 of the 140 cases malleus relocation with malleovestibulopexy was performed using reshaped necrosed incus [16].

**Table 3:** Types of prosthetics used and revised in each publication

Publication	Procedure	Stainless steel/platina teflon piston^a^	Autolog	Malleovestibulopexy	Wire type	Other^b^	Total
E. Vartiainen	Primary		33			12	45
	Revision	43		2			45
W. Han	Primary	31	3		9	6	49
	Revision	–	–	–	–		0
L. Krieger	Primary				26	57	83
	Revision					83	83
W. Lippy	Primary	–	–	–	–	–	0
	Revision	–	–	–	–	–	0
R. Puxeddu	Primary	39			1	4	44
	Revision	34				10	44
G. Babighian	Primary	–	–	–	–	–	0
	Revision	60		5			65
P. Schmid	Primary	–	–	–	–	–	0
	Revision	99	1	64			164
M. Özüer	Primary	–	–	–	–	–	0
	Revision	51		9		6	66
J. Skrivan	Primary	–	–	–	–	–	0
	Revision	33				10	43
M. Ghonim	Primary	–	–	–	–	–	0
	Revision	32		4			36
D. Bernardeschi	Primary	–	–	–	–	–	0
	Revision	–	–	–	–	–	0
U. Fisch	Primary	36		5	8	8	57
	Revision	15		56			71
Total primary		106 (38%)	36 (13%)	5 (2%)	44 (16%)	87 (31%)	278
Total revision		367 (59%)	1 (0%)	140 (23%)	0 (0%)	109 (18%)	617

^a^Classic pistons shape prosthesis

^b^Bucket type, strut type, shape-memory, soft clip, etc.

Regarding the surgical findings, we found relevant data in 846 cases. In the publications sometimes multiple concomitant causes were reported for the same operation. The most common problem reported was necrosis of the long process of incus in 41%, followed by prosthesis dislocation in 36% and periprosthetic scarring in 23%. Tympanic membrane pathologic findings were not mentioned numerically (Table 4).

**Table 4:** Causes of failure reported in each publication

	LPI necrosis	Prosthesis dislocation	Oval window reobliteration/incomplete fenestration	Periprosthetic scarring	Incus luxation, incus/malleus ankylosis	No. of cases
E. Vartiainen	2	11	14	17	1	45
W. Han	32	43	21	37	3	74
L. Krieger	83	0	0	0	0	83
W. Lippy	n	n	n	n	23	23
R. Puxeddu	11	13	8	10	2	44
G. Babighian	25	31	17	12	1	78
P. Schmid	67	107	53	53	16	172
M. Özüer	15	0	7	42	2	66
J. Skrivan	7	12	8	12	4	43
M. Ghonim	36	0	0	0	0	36
D. Bernardeschi	43	31	6	6	16	102
U. Fisch	30	60	36	9	20	80
total	351	308	170	198	88	846
total (%)	41%	36%	20%	23%	10%	

LPI: long process of incus

Separate audiological data in relevant numbers could be found related to two specific situations: cases where malleovestibulopexy was performed and cases where cause of failure was incus necrosis. In case of malleovestibulopexy the data of 129 cases were processed and ABG closure of 10 dB or less, 11–20 dB, and > 20DB was found in 36, 46 and 18% respectively (Table 5a). Concerning incus necrosis according to data of 251 cases ABG closure was 60, 30, and 10% respectively (Table 5b).

**Table 5:** Audiological results in 10 dB bins in case of (a) Malleovestibulopexy (b) Incus necrosis

a	0–10 dB	11–20 dB	> 20 dB	No of cases
G. Babighian	2	2	1	5
P. Schmid	32	22	10	64
M. Ghonim	2	2	0	4
U. Fisch	10	33	13	56
Total	46 (36%)	59 (46%)	24 (18%)	129

b	0–10 dB	11–20 dB	> 20 dB	No of cases
M. Ghonim	28	8	0	34
L. Krieger	53	21	9	83
G. Babighian	17	7	1	25
P. Schmid	53	39	17	109
Total	151 (60%)	75 (30%)	27 (10%)	251

*Complications*: Severe complications were observed in 17 cases (1.6%). All of them were severe SNHL that occurred after surgery, 2 cases had profound hearing loss. Transient taste disturbance (most likely related to injury of the chorda tympani) and transient tinnitus were mentioned. Numerical data for these latter two complications were not available. These mentioned complications completely resolved within 3–6 weeks after surgery. Permanent vertigo was reported in 2 cases. In one additional case, vertigo and disequilibrium occurred one year after the revision, however, in this case further investigation did not prove direct connection with the revision surgery.

## Discussion

### Interpretation of the results

The results of our review suggest that revision stapes surgery is a viable option in cases of failure or relapse after primary surgery. We also found a relevant ABG closure and hearing gain rates, whereas complication rate was about 1.6%. At first glance, the ABG gain of 17.3 dB can be compared with primary stapedotomy results, where the average ABG closure and hearing gain is about 20 dB, with most reports in the range of 18–24 dB [3, 7, 25, 26], still a negative difference of nearly 15%. Complication rates after primary surgery ranged between 0.5 and 2% [3, 27, 28] but were mainly reported below 1%, although the complication rate at revisions was 1.6%. Thus, the probability of developing a permanent adverse situation is about two-fold. The vast majority of these cases are severe SNHL, vestibular symptoms appear to be rare, dysgeusia was not documented in detail in the involved publications. ABG levels are always reported in papers on stapes surgery; however, definitive AC levels and hearing gains are often missing, even in otherwise high-quality papers. Due to the above-mentioned phenomenon, reporting exclusively ABG results after revision surgery can be misleading. In terms of the distribution of the results, looking at the literature we can see comparable outcomes. One of the most cited publications in the topic is Robert Vincent’s Prospective Study of 652 Cases [29], which couldn’t be involved because of our inclusion criteria (inferior age limit was 16 years). In this publication the minimum one year follow up was achieved in 278 cases. The postoperative 10 dB distribution is the following: 10 dB or less 65%, 11–20 dB 9%, > 20 dB 26%. The results at one year follow up (n = 197) was: 10 dB or less 67.5%, 11–20 dB 15.5%, > 20 dB 17%. According to the 875 cases reviewed ABG closure of 10 dB or less was achieved in 53.3%, 10–20 dB 28.2%, > 20 dB 18.5%. The results of Vincent are somewhat above the average of the data analysed in the review.

Comparing this result with primary stapes surgery we could see the following: surgical success (ABG 10 dB or less) was achieved in 53.3% of the revision cases vs. 70 and 94% of primary surgery outcomes [3, 7, 26, 30, 31]. The ABG range of 20 dB or less was achieved in 81.5% of the cases. The same 0–20 dB ABG interval is seen in 92–98% [3, 7, 31] of primary surgeries. In 18.5% of the cases, the ABG remained above 20 dB, which can be considered as an unsuccessful intervention. On the basis of all this information, it is clear that the results and expectations for revision surgeries are not the same as for primary procedures. It has been shown that hearing improvement can be achieved in general, but outcomes are usually more modest compared to primary stapes surgeries. In terms of the distribution of results, the variability of outcomes appears to be greater when revisions are performed. When revisions are compared to primary surgeries, the probability of moderate results (ABG between 10 and 20 dB) is quite similar. The main difference between the upper and the bottom section of results is seen with a range far worse below 10 dB to above 20 dB ratio, which is around 0.35 for revision cases vs. around 0.03–0.1 for primary surgeries [3, 7] in the literature. This means that the postoperative audiological results of ineffective revision surgeries not only fall into the mid-range, but often right into the unsuccessful range. This variability is due to various intratympanic scenarios, influenced by the original pathology, previous surgery, and post-surgical reactions.

According to analysed data, no numerical analysis could be performed regarding the type of retrieved prosthesis and hearing outcome. However, it looks like the former use of older “wire type” prosthesis leads more frequently to incus necrosis [18, 20]. Looking at the prosthetics inserted during revision, classic piston type and other modern prosthetics (e.g. soft clip, shape-memory pistons) are dominating, while autologous and “wire types” are hardly in use anymore. In case of severe incus necrosis, incus luxation or ankylosis malleovestibulopexy is a therapeutical option, which was performed in 23% of the operations. This intervention led to < 20 dB ABG closure in 82%, which is very close to the results of revision surgeries in general. Other results from the literature suggest incudovestibulopexies are superior to malleovestibulopexies from audiological standpoint [21, 33]. Still, the latter is definitely a viable and recommended solution in case of damaged incus for ossicular chain reconstruction.

Certain situations, such as intratympanic scarring, appear to be negative predictors of surgical success [6, 10], whereas prosthesis related problems might be easier to manage [8, 32]. According to the data, in cases of incus necrosis alone without any fibrosis ABG could be closed below 10 dB in 60% and below 20 dB in 90% of the cases. These numbers are nearly 10 percent better than results in revision surgery in general. Thus, incudovestibulopexy should be preferred vs. malleovestibulopexy if it can be performed reasonably.

The technique of the previous surgery also seems to be determinant. Partial and total removal of the stapes footplate has a detrimental effect on outcomes according to literature. It has been reported in certain cases that the long-term AC gain after revision stapedectomy can be as low as 7 dB [8, 32, 34]. All the above-mentioned aspects should be taken into consideration when discussing possible postoperative expectations. Therefore, preoperative workup with HRCT imaging is strongly recommended, even if the evaluation is often challenging [35, 36], and it is not always possible to assess the exact problem underlying the inadequate hearing results.

In none of the included publications was the issue of advanced otosclerosis addressed. According to the review of Adrien A. Eshraghi and his team on this topic [37], stapedotomy alone, promises good results in cases with speech recognition scores higher than 50%, and minor retrofenestral involvement, in contrary cases with major retrofenestral involvement and a speech recognition score less than 30% are proven to have unsatisfactory results. In these latter cases a combination with traditional hearing aids may provide a sufficient solution with measurable hearing improvement and patient contentment. In cases where stapedotomy alone or in combination with traditional hearing aid still do not result in sufficient audiological results, cochlear implantation should be considered, however, electrode insertion may be challenging due to sclerosis and spongiosis. As an alternative in certain cases active middle ear implants may provide an efficient solution in conductive or mixed hearing losses [38, 39]. As in primary surgeries, conventional hearing aids alone could be possible alternatives of revision operations as well.

Concerning the indication of the surgery we have take all these aspects into consideration. Mostly the indications for revision is relative and are based on the possibility of hearing improvement. In general, it is realistic to set 20 dB ABG gain and 17.5 dB AC gain as achievable goal and the indication should be made accordingly. In certain situations, the expectable outcomes are better, like in cases of incus necrosis without malleovestibulopexy. That is why the use of CT scanning could help to predict the most likely outcomes.

## Conclusion

Our review consolidate that different audiological outcomes may be expected after revisions with different causes of failure.

Audiological data suggest that AC gain and ABG gain results were similar after revision stapes surgeries. However, it seems that there is a greater chance of sensorineural threshold shift with repeated interventions, so it would be also useful to assess not only ABG results but also AC results. While ABG is an important indicator in conductive hearing loss, the quality of life is ultimately determined by the AC threshold. This is why we recommend that AC levels and gains should always be reported after revision surgery. Generally speaking, the results are worse compared to primary surgery. A maximum of 10 dB ABG is widely accepted as success after primary stapes surgery. However, as we saw earlier, it is unrealistic to have the same expectations for revisions. While the 10 dB threshold can only be achieved in 53.3%, the range below 20 dB can be achieved in 81.5%. The latter percentage is similar to the success rates (≤ 10 dB) of primary surgery (70–94%). In our opinion, it is realistic to consider the range below 20 dB as a success range after revision stapes surgery. However, if we are talking about individual cases, it is important to assess the preoperative hearing status to set our goals. The mean 17.5 dB AC gain could be another cornerstone of expectations, and achieving this range of AC gain may also be an indicator of surgical success.

## Supplementary Information

Below is the link to the electronic supplementary material.

Supplementary file1 (XLSX 11 KB)

## Data Availability

Not applicable.

## Abbreviations

PRISMA: Preferred Reporting Items for Systematic Reviews and Meta-Analyses
NOS: Newcastle–Ottawa Scale
SNHL: Sensorineural hearing loss
ABG: Air bone ap
AC: Air conduction
BC: Bone conduction
PTA: Pure tone audiometry
RCT: Randomised controlled trial
